# White Matter Pathology as a Barrier to Gangliosidosis Gene Therapy

**DOI:** 10.3389/fncel.2021.682106

**Published:** 2021-08-12

**Authors:** Anne S. Maguire, Douglas R. Martin

**Affiliations:** ^1^Scott-Ritchey Research Center, Auburn University College of Veterinary Medicine, Auburn, AL, United States; ^2^Department of Anatomy, Physiology, and Pharmacology, Auburn University College of Veterinary Medicine, Auburn, AL, United States

**Keywords:** GM1 gangliosidosis, GM2 gangliosidosis, Tay-Sachs disease, Sandhoff disease, AAV gene therapy, white matter, myelin, oligodendrocytes

## Abstract

The gangliosidoses are a family of neurodegenerative lysosomal storage diseases that have recently seen promising advances in gene therapy. White matter deficits are well established components of gangliosidosis pathology that are now receiving more attention because they are partially refractory to correction by gene therapy. After a brief synopsis of normal myelinogenesis, this review outlines current viewpoints on the origin of white matter deficits in the gangliosidoses and potential obstacles to treating them effectively by gene therapy. Dysmyelinogenesis (failure of myelin sheaths to form properly) is proposed as the predominant contributor to white matter pathology, but precise mechanistic details are not well understood. The involvement of neuronal storage deficits may extend beyond secondary demyelination (destruction of myelin due to axonal loss) and contribute to dysmyelinogenesis. Preclinical studies in animal models of the gangliosidoses have substantially improved lifespan and quality of life, leading to the initiation of several clinical trials. However, improvement of white matter pathology has lagged behind other metrics and few evidence-based explanations have been proposed to date. Research groups in the field are encouraged to include myelin-specific investigations in future gene therapy work to address this gap in knowledge.

## Introduction

GM1 and GM2 gangliosidosis (GM1 and GM2) are devastating lysosomal storage diseases that result in the neurodegenerative decline and death of children before 5 years of age in most cases. This family of autosomal recessive diseases causes the dysfunction of the lysosomal enzymes β-galactosidase (βgal) or β-*N*-acetylhexosaminidase (Hex) resulting in the buildup of GM1 or GM2 ganglioside, respectively, in neuron cell bodies. GM1 results from mutation of the *GLB1* gene and affects one in 100,000–200,000 live births (Brunetti-Pierri and Scaglia, 2008). Three clinically similar subtypes of GM2 exist: Tay-Sachs disease (TSD), Sandhoff disease (SD), and GM2 activator deficiency, which result from mutation of *HEXA*, *HEXB*, and *GM2A* genes, respectively. While TSD and SD affect one in 200,000–400,000 live births (Meikle et al., 1999), GM2 activator deficiency occurrence has only been recorded in a handful of cases across the world. Regardless of genetic origin, dysfunction of the βgal or Hex enzymes causes a cascade of central nervous system (CNS) symptoms in affected patients such as delay or loss of developmental milestones, difficulty swallowing, seizures, and ultimately death. Only palliative treatment is available currently, but clinical trials with gene therapy began in 2019. The animal studies that preceded clinical trials have shown dramatic improvement in clinical metrics, but complete normalization has yet to be achieved reproducibly. To maximize patient benefit, and because gene therapy manufacturing is expensive and labor-intensive, it is crucial to make sure that each dose is as effective as possible.

**FIGURE 1:**
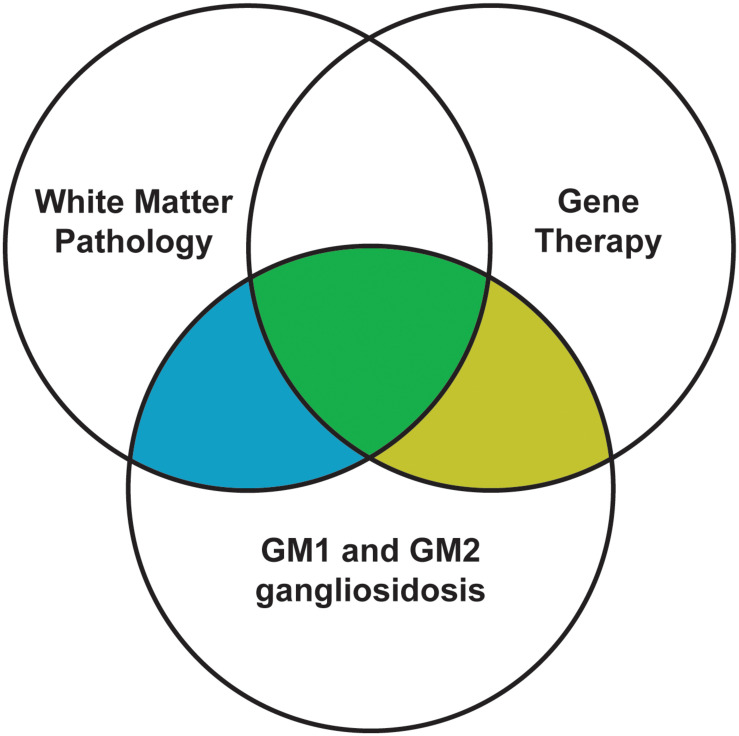
Scope of this review. Filled areas are included in this review, while unfilled areas are covered elsewhere.

While gene therapy usually results in enzyme activity at or above effective levels (10–20% of normal) and substantially reduced neuronal storage, lifespans and other measures of success are still not at normal levels. This leads researchers to suspect that other deficits persist despite gene therapy, such as inflammation and myelin pathology. Indeed, white matter deficits are increasingly considered therapeutic targets in neurodegenerative diseases such as Alzheimer’s disease (Nasrabady et al., 2018), Parkinson’s disease (Luo et al., 2017), and other closely related lysosomal storage diseases (Takikita et al., 2004; Buccinnà et al., 2009; Provenzale et al., 2015). Similarly, white matter pathology in the gangliosidoses has been characterized since the 1970s and is now receiving attention as a phenomenon potentially separate from gray matter deficits. While early gene therapy efforts focused on enzyme activity restoration and storage reduction, recent studies have implied that myelin pathology is at least partially refractory to gene therapy. Therefore, to improve the efficacy of treatment, the field would benefit from future gene therapy studies incorporating white matter metrics. After a brief synopsis of normal myelinogenesis, this review encompasses three overlapping areas of study in the gangliosidoses (Figure 1): white matter pathology, the current state of gene therapy success, and the effect of gene therapy on white matter pathology.

### Normal Myelinogenesis

Normal myelinogenesis involves three major processes: oligodendrocyte development, myelin sheath assembly, and axonal communication. Oligodendrocytes, the glial cells responsible for myelinating axons within the CNS, have four developmental stages: oligodendrocyte precursor cell (OPC), preoligodendrocyte, immature oligodendrocyte, and mature oligodendrocyte (Barateiro and Fernandes, 2014). Common protein markers used for identification of these stages include NG2 (neural/glial antigen 2) and PDGF-Rα (platelet-derived growth factor receptor α) for OPCs and preoligodendrocytes, with the additional expression of O4 and GRP17 marking a transition between the two cell types. O4 expression is retained by immature oligodendrocytes, which also produce GalC (galactocerebroside) and CNPase (2′,3′-cyclic nucleotide 3′-phosphodiesterase). MBP (myelin basic protein), PLP (proteo-lipid protein), and MAG (myelin-associated glycoprotein) are markers of mature oligodendrocytes (Barateiro and Fernandes, 2014). In humans, this process is initiated at 10 weeks post-conception when OPCs first appear, and it reaches completion perinatally with the appearance of substantial numbers of MBP-positive mature oligodendrocytes (Barateiro and Fernandes, 2014).

Myelin sheath components include specific families of lipids in addition to the proteins present on the mature oligodendrocyte cell membrane (such as MBP and PLP). In humans, the appearance of these substances follows a prescribed sequence consisting of major lipids and proteins that can be detected biochemically. Cholesterol synthesized by oligodendrocytes is a major component and critical precursor of myelin sheaths, as well as a rate-limiting factor in their formation (Saher et al., 2005). Formation of phospholipids precedes the emergence of sphingomyelin, which is followed by the near-simultaneous appearance of cerebrosides, sulfatides, MBP and PLP. The latter four substances are the primary components of adult myelin (Kinney et al., 1994). The onset of this sequence varies substantially between anatomical regions, but ranges from mid-gestation (for the tracts that are the earliest to myelinate) to early infancy (for the later-developing tracts) (Kinney et al., 1994).

Recent evidence has revealed that neuroaxonal communication with oligodendrocytes and myelin sheaths is critical to the development of mature myelin and the maintenance of plastic neuronal circuitry (Almeida and Lyons, 2017; Chorghay et al., 2018; Turner, 2019). Indeed, emerging concepts such as activity-dependent myelination and the existence of an axomyelinic synapse (as reviewed in Chorghay et al., 2018) imply that axonal interactions must be considered when investigating oligodendrocyte and myelin development. For example, glutamate released from axons after action potentials likely binds to receptors on both OPCs and oligodendrocytes, with the former hastening OPC maturation and the latter facilitating myelin sheath plasticity and maintaining CNS circuitry (Chorghay et al., 2018). Still undergoing active research is the timing of activity-dependent myelination mechanisms, which appear to be highly variable throughout brain regions and developmental experiences. A detailed understanding of this concept would provide invaluable insight into the pathology of diseases, such as the gangliosidoses, with concurrent developmental and degenerative components.

## White Matter Pathology in the Gangliosidoses

**FIGURE 2:**
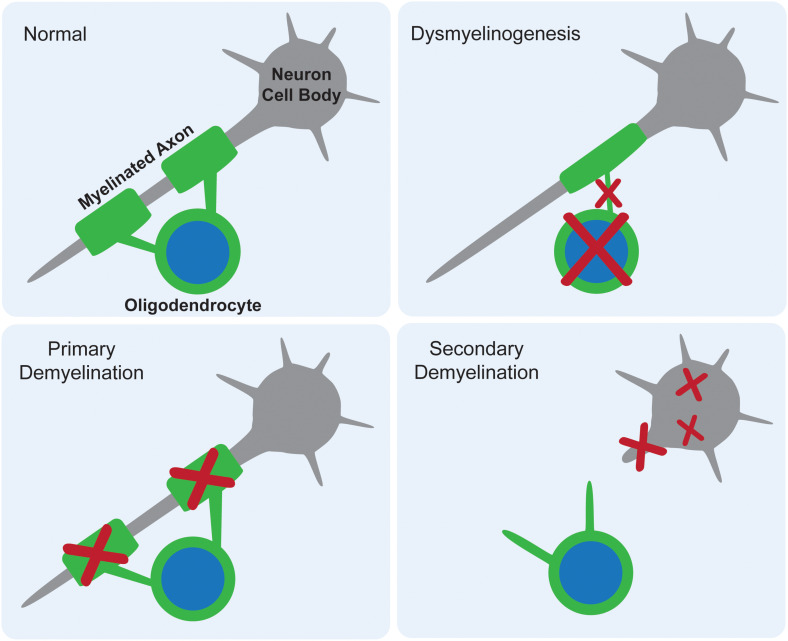
Proposed mechanisms for myelin deficits in the central nervous system. In normal tissue, each oligodendrocyte modifies its own cell membrane to myelinate axons. If dysmyelinogenesis occurs, pathology within the oligodendrocyte, early myelin structures, or neuroaxonal communication leads to an abnormally myelinated axon. In primary demyelination, the oligodendrocyte is functional and myelin is formed properly, but then degraded. In secondary demyelination, neuroaxonal dysfunction leads to axonal loss, resulting in the overall loss of myelinated axons.

White matter deficits have long been reported in the gangliosidoses, with occasional speculation as to their origin. There are three proposed mechanisms for how myelin deficits occur as part of any CNS pathology: dysmyelinogenesis (failure to form properly), primary demyelination (destruction after proper formation), or secondary demyelination (loss after axonal degeneration) (Figure 2). An emerging theme in the gangliosidosis literature suggests that dysmyelinogenesis is the main contributor to white matter pathology, with primary and secondary demyelination playing less important roles. Last reviewed by Folkerth (1999), this hypothesis is supported by early deficits in white matter tract development and abnormal myelin sheath structure. A detailed understanding of the mechanism for myelin pathology remains elusive in the current literature, with evidence available to support interference with all major components of myelinogenesis: oligodendrocytes, myelin sheaths, and neuroaxonal complexes. The use of inducible mouse models of gangliosidosis, which permit shutdown of Hex expression at different ages, may provide insight into myelin pathogenesis at critical developmental stages (Sargeant et al., 2012).

### Early Deficits in White Matter Tract Development

Though a generalized “loss of white matter” is routinely reported as part of the diagnostic process in case studies of the gangliosidoses, several publications examine these deficits in more detail to elucidate the timing of the onset of dysmyelinogenesis. The most common methods include magnetic resonance imaging (MRI) and histochemical staining (Weil or Luxol Fast Blue). In cat, dog, and sheep models of both GM1 and GM2, cerebellar and cerebral cortical white matter is universally considered decreased compared to normal counterparts (Kaye et al., 1992; Kroll et al., 1995; Porter et al., 2011; Gray-Edwards et al., 2020), while results for non-cortical white matter are mixed. In GM1 (Folkerth et al., 2000) and GM2 (Haberland et al., 1973) patients, tracts that would normally develop prenatally are less affected than tracts that develop postnatally, so a perinatal onset of dysmyelinogenesis is hypothesized.

### Abnormal Myelin Sheath Architecture

The myelin sheath in GM1 and GM2 has been critically evaluated from two perspectives: quantification of its major components and evidence of its breakdown (Table 1). The most thoroughly investigated myelin sheath components are cerebrosides and sulfatides, which have been shown *via* high performance thin layer chromatography (HPTLC) to be decreased in CNS tissue across mice, dogs, cats, and human patients with both GM1 and GM2 (Haberland et al., 1973; Kaye et al., 1992; Chakrabarti et al., 1996; Broekman et al., 2007; Baek et al., 2009, 2010; Heinecke et al., 2015; Rockwell et al., 2015; Table 1). Collectively, these studies implicate that such deficits occur in most major regions of the CNS. A notable exception was observed in Gray-Edwards et al. (2017a), who found an increase in cerebrosides and sulfatides in cerebrospinal fluid (CSF) of cats with GM1, potentially indicating leakage due to myelin breakdown in tissue.

**TABLE 1:** Trends in pathological changes to oligodendrocytes, axons, and myelin in GM1 and GM2 gangliosidosis.

	Myelin			Oligodendrocytes			Axons			
	Cerebrosides/sulfatides	Sheath integrity	Protein/mRNA markers	Storage	Number	Cell injury	Degenerative changes	Axon number	Cell body number	Number myelinated
**GM1**										
Human		NC^1^↓^1^	↓^2^	NC^1–3^	↓^1,2^	↑^2^	NC^1,2^	↓^2^		↓^1,2^
Mouse	↓^4–6^	↓^4^								
Cat	↑^7^*	↓^8^		NC^8^	NC^8^	↑^8^				
Dog	↓^9^						↑^9^			↓^9^
**GM2**										
Human	↓^3,10,11^	↓^10^			NC^10^		↑^10^			↓^10^
Mouse	↓^3^		↓^12^				NC^13^↑^12^		↓^12,14^	
Cat	↓^3,15^	NC^16^					↑^16^	NC^16^		NC^16^
Sheep							↑^17^			
Dog		NC^18^		↑^18^			↑^18,19^		NC^18^	

*Measured cerebrosides/sulfatides in CSF rather than CNS parenchyma. ↑, increased from normal; ↓, decreased from normal; NC, no change from normal. References: ^1^Folkerth et al., 2000, ^2^van der Voorn et al., 2004, ^3^Baek et al., 2009, ^4^Heinecke et al., 2015, ^5^Broekman et al., 2007, ^6^Baek et al., 2010, ^7^Gray-Edwards et al., 2017a, ^8^Gray-Edwards et al., 2020, ^9^Kaye et al., 1992, ^10^Haberland et al., 1973, ^11^Sandhoff et al., 1971, ^12^Cachón-González et al., 2014, ^13^Walkley, 2000, ^14^Sargeant et al., 2011, ^15^Rockwell et al., 2015, ^16^Kroll et al., 1995, ^17^Porter et al., 2011, ^18^Cummings et al., 1985, and ^19^Sanders et al., 2013.

The consensus on cerebroside and sulfatide deficiencies in GM1 and GM2 leads to the question of whether there are similar changes in the major myelin proteins that emerge at the same time in normal myelinogenesis. Unfortunately, only two studies have investigated protein and mRNA levels for MBP, PLP, MAG, and CGT (UDP-galactose:ceramide galactosyltransferase), and more region-specific work is needed to draw concrete conclusions about the onset of white matter pathogenesis. For example, protein levels of MBP and PLP are decreased both in the frontal lobe of humans with GM1 (van der Voorn et al., 2004) and the cerebrum of mice with GM2 (Cachón-González et al., 2014), but no other regions have undergone quantitative analysis. The mRNA levels of MBP, MAG, and CGT in the olfactory bulb, cerebrum, cerebellum, brainstem, and spinal cord of GM2 mice were either at or below normal levels, with the only consistencies across all three markers occurring in the cerebrum (normal levels) or brainstem (below normal levels) (Cachón-González et al., 2014). Future studies should examine whether a correlation exists between protein/mRNA levels and specific white matter tract deficiencies that have previously been established (Folkerth, 1999).

Histological and ultrastructural assessments of myelin sheath integrity inconsistently note pathological changes (Table 1), but animal model studies only examine one or two CNS regions at this level (Cummings et al., 1985; Kroll et al., 1995; Heinecke et al., 2015; Gray-Edwards et al., 2020). More informative are the comprehensive evaluations of autopsy cases of GM1 (Folkerth et al., 2000) and GM2 (Haberland et al., 1973) patients that compared major CNS regions and white matter tracts. Both studies noted abnormally thin sheaths that were otherwise structurally intact. In GM2 patients, sudanophilic breakdown products were found throughout the CNS in a pattern that roughly corresponded with neuronal loss (Haberland et al., 1973). These breakdown products were presumed to be degenerating myelin and axons, prompting the proposed mechanism that secondary demyelination occurs after neuronal loss caused by storage or metabolic insults (Haberland et al., 1973).

In conclusion, the evidence for early deficiencies in the major components of myelin with few indications of their destruction supports the hypothesis that dysmyelinogenesis is the predominant mechanism of white matter pathology, with minor contributions from primary and/or secondary demyelination. This conclusion could be modified as more investigations are conducted into myelin proteins and sheath degeneration across CNS regions in animal models.

### Oligodendrocyte Pathology

Pathological changes to oligodendrocytes have been found across species (Table 1), and CNS regions. Oligodendrocyte number is qualitatively decreased upon evaluation of limited regions of GM1 human tissue (Folkerth et al., 2000; van der Voorn et al., 2004), but unchanged from normal levels when immunohistochemistry (IHC) staining is quantified throughout the brain of GM1 cats (Gray-Edwards et al., 2020). The only study to investigate oligodendrocytes in GM2 found a similar number in affected human patients compared to normal (Haberland et al., 1973). Evidence of oligodendrocyte cell injury is apparent in GM1 through TUNEL staining (van der Voorn et al., 2004) or ultrastructural examination (Gray-Edwards et al., 2020). Despite these abnormalities in number and pathological changes, the consensus appears to be that ganglioside storage does not occur in oligodendrocytes (Folkerth et al., 2000; van der Voorn et al., 2004; Baek et al., 2009; Gray-Edwards et al., 2020), with the only exception reported in one Japanese spaniel with GM2 (Cummings et al., 1985). Authors that observe oligodendrocyte pathology consider it to play a substantial role in the development of white matter deficits, but specific mechanisms of action and the extent of concurrent dysfunctional neuroaxonal communication have yet to be delineated.

### Neuroaxonal Dystrophy

Neuronal pathology incited by ganglioside storage likely contributes to myelin pathology through deficiencies in axonal-oligodendrocyte communication, secondary demyelination, or both. While the separate concepts of neuroaxonal dystrophy in GM1/GM2 (Table 1) and axon-led activity-dependent myelination (Chorghay et al., 2018) are well-established in the literature, they have not been thoroughly investigated together as a potential mechanism for white matter deficits in the gangliosidoses. Common methods for investigating neuroaxonal pathology include quantitative IHC (NeuN stain), and qualitative evaluation of hematoxylin and eosin (H&E) staining, silver staining, and ultrastructure. In most species with gangliosidosis, myelinated and total numbers of axons are decreased (Haberland et al., 1973; Kaye et al., 1992; Folkerth et al., 2000; van der Voorn et al., 2004). This could be due to degenerative changes within axons, which are inconsistently found throughout the CNS (Haberland et al., 1973; Cummings et al., 1985; Kaye et al., 1992; Kroll et al., 1995; Folkerth et al., 2000; Walkley, 2000; van der Voorn et al., 2004; Porter et al., 2011; Sanders et al., 2013; Cachón-González et al., 2014), or secondary to the decrease in cell body number noted in mice with GM2 (Sargeant et al., 2011; Cachón-González et al., 2014). Regardless of cause, the contribution of axonal loss to secondary demyelination and/or dysfunctional activity-dependent myelination represents an intriguing area of future study.

## Gene Therapy in the Gangliosidoses

Gene therapy for GM1 and GM2 has achieved outstanding preclinical results using several vector backbones, capsids, delivery routes, and animal models (Table 2). Full reviews of the status of gene therapy in the gangliosidoses have recently been published elsewhere (Bradbury et al., 2015b; Kelly et al., 2017; Rha et al., 2021). Initial studies in the gangliosidoses involved lentivirus and adenovirus (Guidotti et al., 1999; Bourgoin et al., 2003; Takaura et al., 2003; Kyrkanides et al., 2005, 2007), but many recent studies focus on adeno-associated virus (AAV). Intracranial administration sites included the striatum or thalamus with or without the cerebellum (Cachon-Gonzalez et al., 2006; Baek et al., 2010; Sargeant et al., 2011; Cachón-González et al., 2012, 2014; Bradbury et al., 2013; McCurdy et al., 2014, 2015), structures with important functional roles and generally well connected with other regions of the CNS. Concerns about the risk of cerebellar surgery in affected children led to studies that substituted the lateral ventricle [intracerebroventricular (ICV)] for cerebellum injection sites (Rockwell et al., 2015; Gray-Edwards et al., 2018, 2020). Recently, injections of the cisterna magna and/or intravenous routes have shown promising results and led to clinical trials in humans. Taghian et al. (2020) describe a novel technique for administering vector intrathecally in two TSD patients as part of an expanded access trial. Also, a phase I/II clinical trial for GM2 gangliosidosis (ClinicalTrials.gov Identifier NCT04669535) and three trials for GM1 children are underway or almost so (NCT03952637; NCT04713475; and NCT04273269).

**TABLE 2:** Studies involving animal models treated with gene therapy.

First Author	Year	Disease	Species	Route	Vector
Guidotti^1^	1999	GM2 (TSD)	Mouse	Intravenous, intramuscular	Adenovirus
Takaura^2^	2003	GM1	Mouse	Intravenous	Adenovirus
Bourgoin^3^	2003	GM2 (SD)	Mouse	Intracranial (intracerebral)	Adenovirus
Kyrkanides^4^	2005	GM2 (SD)	Mouse	Intraperitoneal	Lentivirus
Cachon-Gonzalez^5^	2006	GM2 (SD)	Mouse	Intracranial (striatum)	AAV1
Kyrkanides^6^	2007	GM2 (SD)	Mouse	Intraperitoneal	Lentivirus
Broekman^7^	2007	GM1	Mouse	Intracranial (ICV)	AAV1
Broekman^8^	2009	GM1	Mouse	Intracranial (hippocampus)	AAV1
Baek^9^	2010	GM1	Mouse	Intracranial (thalamus and DCN)	AAV1
Sargeant^10^	2011	GM2 (SD)	Mouse	Intracranial (striatum)	AAV1
Cachon-Gonzalez^11^	2012	GM2 (SD)	Mouse	Intracranial (striatum, hippocampus, and cerebellum)	AAV1
Bradbury^12^	2013	GM2 (SD)	Cat	Intracranial (thalamus)	AAV1, rh8
Cachon-Gonzalez^13^	2014	GM2 (SD)	Mouse	Intracranial (striatum and cerebellum)	AAV1
McCurdy^14^	2014	GM1	Cat	Intracranial (thalamus and DCN)	AAV1, rh8
McCurdy^15^	2015	GM2 (SD)	Cat	Intracranial (thalamus and DCN)	AAVrh8
Rockwell^16^	2015	GM2 (SD)	Cat	Intracranial (thalamus and ICV)	AAVrh8
Weismann^17^	2015	GM1	Mouse	Intravenous	AAV9
Walia^18^	2015	GM2 (SD)	Mouse	Intravenous	AAV9
Gray-Edwards^19^	2015	GM2 (SD)	Cat	Intracranial (thalamus, DCN, and ICV)	AAV1, rh8
Bradbury^20^	2015	GM2 (SD)	Cat	Intracranial (thalamus)	AAVrh8
Tropak^21^	2016	GM2 (SD)	Mouse	Intravenous	AAV9
Bradbury^22^	2017	GM2 (SD)	Cat	Intracranial (thalamus, DCN, and ICV)	AAV1, rh8
Gray-Edwards^23^	2017	GM1	Cat	Intracranial (thalamus and DCN)	AAV1, rh8
Gray-Edwards^24^	2017	GM1	Cat	Intracranial (thalamus and DCN)	AAVrh8
Gray-Edwards^25^	2018	GM2 (TSD)	Sheep	Intracranial (thalamus and ICV)	AAVrh8
Gray-Edwards^26^	2020	GM1	Cat	Intracranial (thalamus and ICV)	AAVrh8
Taghian^27^	2020	GM2 (TSD)	Sheep/Human	Intrathecal (CM)	AAV9
Lahey^28^	2020	GM2 (SD)	Mouse	Intravenous	AAV9, PHP.B
McCurdy^29^	2021	GM2 (SD)	Cat	Intracranial (thalamus and DCN)	AAVrh8

*SD, Sandhoff disease; TSD, Tay-Sachs disease; ICV, intracerebroventricular; DCN, deep cerebellar nuclei; CM, cisterna magna; AAV, adeno-associated virus. References: ^1^Guidotti et al., 1999, ^2^Takaura et al., 2003, ^3^Bourgoin et al., 2003, ^4^Kyrkanides et al., 2005, ^5^Cachon-Gonzalez et al., 2006, ^6^Kyrkanides et al., 2007, ^7^Broekman et al., 2007, ^8^Broekman et al., 2009, ^9^Baek et al., 2010, ^10^Sargeant et al., 2011, ^11^Cachón-González et al., 2012, ^12^Bradbury et al., 2013, ^13^Cachón-González et al., 2014, ^14^McCurdy et al., 2014, ^15^McCurdy et al., 2015, ^16^Rockwell et al., 2015, ^17^Weismann et al., 2015, ^18^Walia et al., 2015, ^19^Gray-Edwards et al., 2015, ^20^Bradbury et al., 2015a, ^21^Tropak et al., 2016, ^22^Bradbury et al., 2017, ^23^Gray-Edwards et al., 2017b, ^24^Gray-Edwards et al., 2017a, ^25^Gray-Edwards et al., 2018, ^26^Gray-Edwards et al., 2020, ^27^Taghian et al., 2020, ^28^Lahey et al., 2020, and ^29^McCurdy et al., 2021.*

The degree of lifespan extension in AAV-treated animals often correlates with other metrics used to evaluate gene therapy success. Common avenues of investigation include enzyme activity, ganglioside storage, lipid content, inflammatory response, and other histopathological changes. These assessments are also often used to investigate potential mechanisms of gangliosidosis pathogenesis. Recently, biomarkers such as neurological status, MRI, MRS, CSF, and blood chemistry markers have emerged as valuable tools for assessing treatment effectiveness in-life (Bradbury et al., 2015a; Regier et al., 2016; Gray-Edwards et al., 2017b). Some of these biomarkers correlate well between human patients and animal models, and are therefore expected to play important roles in clinical trials (Regier et al., 2016).

## Gene Therapy and Myelin in the Gangliosidoses

Though supporting evidence is needed, investigations into white matter deficits within gene-therapy treated animal models of the gangliosidoses imply that myelin deficits are refractory to gene therapy treatment. Cachón-González et al. (2014) found that myelin protein content in treated mice remained abnormally low regardless of age at treatment and petitioned for further investigation with a larger cohort of animals. However, early treatment seemed to provide the best opportunity to preserve myelin. SD mice treated at 12 weeks old consistently demonstrated MBP and PLP at levels 40% of normal, while those treated at 4 and 8 weeks old showed higher levels, albeit with high variability between animals (Cachón-González et al., 2014). Similarly, treatment age of SD cats was inversely correlated with survival, with those treated early in the post-symptomatic period faring better than those treated late (lifespan 3.5 and 1.5 times that of untreated SD cats, respectively) (McCurdy et al., 2021). Gray-Edwards et al. (2020) found that AAV treatment only partially corrected pale LFB staining and myelin integrity loss evident on TEM. A surprising result was that oligodendrocyte number was not abnormally low in affected untreated animals, but increased in cats treated with AAV, while ultrastructural oligodendrocyte pathology was partially corrected. This leads to an intriguing hypothesis that treated animals produce more oligodendrocytes to compensate for early pathology, which could be further explored through cell death assays specific to oligodendrocytes. Though more myelin-specific work is needed in future gene therapy studies, current evidence indicates that myelin deficits occur before the typical age of AAV treatment or that AAV is ineffective at treating the underlying cause of myelin deficits. One explanation may be that oligodendrocytes are poorly treated by most AAV serotypes (Powell et al., 2016).

It is interesting to consider whether certain routes of AAV delivery are better able to treat myelin pathology than others. While no controlled experiments of myelin preservation have been performed to compare delivery routes, results from feline studies show that myelin integrity generally reflects the overall efficacy of the treatment. For example, to date SD cats have been treated most effectively by injection of the thalamus and deep cerebellar nuclei (DCN) or thalamus and lateral ventricle, with both routes achieving similar increases in survival (∼4.5 times greater than untreated). Animals treated by these routes also had similar preservation of white matter on anatomical MRI, and cerebroside levels in various regions of the brain were similar (McCurdy et al., 2015; Rockwell et al., 2015; unpublished data). Studies are underway in both the SD and GM1 cat models to directly compare intravenous versus CSF-based delivery methods.

While white matter-specific experiments are more informative, insight into how gene therapy affects myelin pathology can be obtained through indirect methods. Baek et al. (2010) found that cerebroside and sulfatide levels in AAV-treated mice were not fully normalized, which implicates early involvement of myelin structural components. *In vivo* MRS measurements of GPC + PCh, an indicator of membrane turnover, were almost completely normalized in AAV-treated cats, which could indicate that refractory pathology does not involve the increased turnover of myelin sheaths (Gray-Edwards et al., 2017b, 2020). Finally, the contribution of secondary demyelination can be indirectly measured through neuron density, which appears to be well-corrected by AAV treatment of SD mice (Sargeant et al., 2011; Cachón-González et al., 2014). These findings implicate an early myelin-specific pathological mechanism that is relatively independent of the loss of neurons and axons.

## Discussion

In conclusion, a few investigations have implied that white matter pathology in the gangliosidoses is refractory to gene therapy. Promising avenues of investigation include oligodendrocyte cell death assays and protein, mRNA, and IHC quantification. Separation of white and gray matter to increase sensitivity to myelin-specific changes is recommended. Though gene therapy has achieved substantial success in improving lifespan, enzyme activity, and other metrics of success, current evidence implies that persistent white matter pathology begins prior to the age of typical gene therapy treatment. This is supported by evidence that the principal mechanism of myelin pathology in the gangliosidoses is dysmyelinogenesis, that likely occurs perinatally. Performing more myelin-centric assays will allow future gene therapy strategies to be refined to correct early-onset refractory white matter deficits. Should it be confirmed that early white matter defects hinder therapy of the gangliosidoses, this will further support the consensus across most lysosomal diseases that early treatment is crucial for long-term benefit. Early diagnosis through programs such as newborn screening will be a critical component of an optimal treatment strategy.

## Author Contributions

AM and DM contributed to the conception of the review. AM wrote the first draft of the manuscript and created the tables and figures. Both authors revised the manuscript and approved the submitted version.

## Conflict of Interest

DM reports stock options from Lysogene and personal fees from Axovant outside the submitted work. The remaining author declares that the research was conducted in the absence of any commercial or financial relationships that could be construed as a potential conflict of interest.

## Publisher’s Note

All claims expressed in this article are solely those of the authors and do not necessarily represent those of their affiliated organizations, or those of the publisher, the editors and the reviewers. Any product that may be evaluated in this article, or claim that may be made by its manufacturer, is not guaranteed or endorsed by the publisher.
